# 

**Published:** 1903-11

**Authors:** 


PUBLISHED MONTHLY

### ATLANTA JOURNAL-RECORD OF MEDICINE.

Successor to Atlanta Medical and Surgical Journal, Established 1855, and Southern Medical Record, Established 1870.

Vol. V. Atlanta, Ga., November, 1903. No. 8.

